# Risk of progression of early-stage mycosis fungoides, 10-year experience^⋆^

**DOI:** 10.1016/j.abd.2023.08.008

**Published:** 2024-02-22

**Authors:** Santiago Andrés Ariza Gómez, Paula Alejandra Dubeibe Abril, Oscar Enrique Niebles Sincelejo, Henry Santiago Leal Reina

**Affiliations:** Dermatology Department, University Foundation of Health Sciences, San José Hospital, Bogotá, Colombia

**Keywords:** Disease progression, Latin America, Lymphoma, T-Cell, cutaneous, Mycosis Fungoides, Prognosis

## Abstract

**Background:**

Mycosis fungoides is the most frequent form of cutaneous T-cell lymphoma. It is characterized by a chronic, slow, and progressive course, and is associated with mortality rates that depend on several factors, such as clinical staging. A median survival time of up to 13 months is found in patients with advanced stages that require more aggressive treatments, with greater toxicity and higher costs. In Latin America, few prognostic studies of the disease are available.

**Objective:**

To determine the rate of progression from early stages (IA, IB, IIA) to more advanced stages (> IIB) in patients older than 18 years with mycosis fungoides treated at two medical centers in Colombia between January 1, 2010, and December 31, 2019.

**Methods:**

Retrospective cohort study with a longitudinal design.

**Results:**

112 patients diagnosed with early mycosis fungoides were included. 56.2% were male (n = 63), with a median age of 53 years (IQR 43‒67). The most frequent clinical variant was classic (67.9%; n = 76), followed by folliculotropic (16%; n = 18), and hypopigmented (10.7%; n = 12). The most common first-line treatment was NB-UVB phototherapy (27.7%; n = 31), followed by PUVA phototherapy (25.8%; n = 29%), and topical corticosteroids (25%; n = 28). The global rate of disease progression was 8% (n = 9), with an overall mortality of 12.5% (n = 14).

**Study limitations:**

Its retrospective design and the lack of molecular studies for case characterization.

**Conclusions:**

Early mycosis fungoides is a disease with a good prognosis in most patients, with a progression rate of 8% (n = 9).

## Introduction

Mycosis Fungoides (MF) is the most common type of cutaneous T-cell lymphoma and accounts for almost 50% of all lymphomas that arise primarily in the skin^1^ and 4% of all non-Hodgkin's lymphomas.^2^ It is characterized by a chronic, slow and progressive course in the early stages. This disease has been associated with a negative impact on quality of life^3^ and a mortality rate that depends on several factors, particularly clinical staging.^4^

Patients with early MF (stages < IIA) usually have a good prognosis, but it has been described that approximately 25% of these patients may progress to advanced forms of the disease (stages > IIB),^5^ presenting with tumors, erythroderma and lymph node, leukemic and/or visceral involvement.^4^ In these cases, median survival is less than 4 years and only 13 months in patients with lymph node involvement,^4^, ^5^ which means that more aggressive, more toxic and costly treatments are required.^6^

Few studies including a large number of cases and reporting the rate of progression and clinical factors affecting the survival of the disease have been published in Latin America. Timely identification of patients with MF who progress is an opportunity to offer more effective and individualized treatments that allow better long-term disease control.

## Methods

A retrospective, multicenter, cohort study, with an analytical approach, which aims to evaluate the rate of progression in patients with mycosis fungoides from early stages (IA, IB, and IIA) to advanced stages (IIB onwards), who were treated at the Hospital San José and at a private dermatology oncology center in Bogotá D.C. during a 10-year period, from January 1, 2010, to December 31, 2019.

Approval was obtained from the institutions' ethics and research committees, and the following inclusion and exclusion criteria were considered:

Inclusion criteria:

• Patients with a skin biopsy-confirmed diagnosis of mycosis fungoides assessed in the dermatology office of Hospital San José and in a private dermato-oncology practice.
• Patients with mycosis fungoides, stages IA, IB, and IIA, according to the classification proposed by the ISCL/EORTC at the time of the initial evaluation.
• Patients over 18 years of age.

Exclusion criteria:

• Lack of relevant data in clinical records.

The TNMB classification proposed by the International Society of Cutaneous Lymphomas (ISCL) and the European Organization for Research and Treatment of Cancer (EORTC), endorsed by the National Comprehensive Cancer Network (NCCN), was used for patient staging. With this system, MF severity is classified into nine stages (IA, IB, IIA, IIB, IIIA, IIIB, IVA1-2, and IVB).^3^, ^4^, ^5^

Similarly, the sociodemographic and clinical characterization of the population was carried out, and possible associated poor prognostic factors described in the literature were identified, namely, advanced age, nodal involvement, folliculotropism, lack of CD7 cells in neoplastic cells, CD30+ expression, large cell transformation, increased LDH (lactate dehydrogenase), eosinophilia in blood count, TCRγδ phenotype, among others.^3^, ^5^ Response to the different lines of treatment offered, as defined by the EORTC and the United States Cutaneous Lymphoma Consortium (USCLC), was also assessed as follows:

- Complete: 100% response (disappearance of all lesions)
- Partial: Response between 50%‒99%
- Null: Response <50%

This was done by collecting qualitative and quantitative variables from the medical records of each patient. Absolute and relative frequency analysis using tables and graphs was used for qualitative variables, whereas measures of central tendency (mean, median), measures of dispersion (standard deviation, interquartile range), and measures of order (percentiles) were used for quantitative variables.

## Results

From January 1, 2010, to December 31, 2019, a total of 214 patients suspected of having mycosis fungoides were evaluated, 137 (64%) at Hospital San Jose, and the remainder at a private dermato-oncology center. Of these, 112 cases (52%) had a confirmed diagnosis of mycosis fungoides and met all the other inclusion criteria.

Overall, 56.2% were male (n = 63), and the median age was 53 (IQR 43‒67) (Table 1). The most common phototype identified was phototype 3. The median progression time of the disease was 62 months (IQR 24‒120) (Table 2). During the initial assessment, semiological lesions were classified as a patch in 42% (n = 47), plaque in 33% (n = 37), and macula in 14.3% (n = 16) of cases. Classic was the most common variant typed at 67.9% (n = 76), followed by folliculotropic at 16% (n = 18), and hypopigmented at 10.7% (n = 12). Regarding stages, IA was the most frequent at 64.3% (n = 72), followed by IB at 25.9% (n = 29) (Table 3).

**Table 1:** Sociodemographic characteristics.

Variable	Value
**Age in years, median (interquartile range)**	53 (43‒67)
**Sex, frequency (percentage)**	
Male	63 (56.2)
Female	49 (47.4)
**Occupation, frequency (percentage)**	
Unknown	20 (17.86)
Household	16 (14.29)
Retired	13 (11.61)
Tradesperson	7 (6.25)
Lawyer	5 (4.46)
Employee	5 (4.46)
Student	5 (4.46)
Teacher	4 (3.57)
Engineer	4 (3.57)
Economist	3 (2.68)
Administrator	2 (1.79)
**Origin, frequency (percentage)**	
Bogotá	78 (69.64)
Cundinamarca	11 (9.82)
No data	9 (8.04)
Boyacá	6 (5.36)
Huila	2 (1.79)
Quindío	2 (1.79)
Tolima	2 (1.79)
Meta	1 (0.89)
Venezuela	1 (0.89)
**Insurance plan, frequency (percentage)**	
Contributive	72 (64.29)
Prepaid	31 (27.68)
Private	9 (8.04)

Note: Data collected by author in 2021.

**Table 2:** Phototype, time of disease progression, and delay in diagnosis.

Variable	Value
**Fitzpatrick skin phototype, frequency (percentage)**	
No data	82 (73.21)
3	24 (21.43)
4	4 (3.57)
2	1 (0.89)
6	1 (0.89)
**Time of progression to first assessment in months, frequency (percentage)**	62 ( 24‒120)
**Year of diagnosis confirmation, frequency (percentage)**	
2015	11 (9.82)
2016	19 (16.96)
2017	13 (11.61)
2018	8 (7.14)
**Time to diagnosis in months, median (IQR)**	60 ( 24‒96)

Note: Data collected by author in 2021.

**Table 3:** Clinical characteristics in the initial vs. final assessment.

Variable		Frequency (percentage) Initial assessment	Frequency (percentage) Final assessment	Likelihood^a^
**Clinical presentation**	Patch	47 (41.96)	37 (33.04)	**0.001**
	Plate	37 (33.04)	18 (16.07)	
	Macule	16 (14.29)	14 (12.5)	
	Papule	4 (3.57)	3 (2.68)	
	Tumor	3 (2.68)	8 (7.14)	
	No data	3 (2.68)	26 (23.21)	
**Clinical variant**	Classic	76 (67.86)	61 (54.46)	**0.001**
	Folliculotropic	18 (16.07)	11 (9.82)	
	Hypopigmented	12 (10.71)	6 (5.36)	
	No data	4 (3.57)	31 (27.68)	
	Erythrodermic	2 (1.79)	2 (1.79)	
	Hyperkeratotic	0 (0.00)	1 (0.89)	
**Disease stage**	IA	72 (64.29)	55 (49.11)	**0.001**
	IB	29 (25.89)	4 (12.5)	
	IIA	1 (0.89)	0 (0.0)	
	IIB	0 (0.0)	11 (9.82)	
	III	0 (0.0)	1 (0.89)	
	IIIB	0 (0.0)	1 (0.89)	
	No data	10 (8.92)	30 (26.78)	

Note: Data collected by author in 2021.

a Chi-Squared.

In the last follow-up visit, clinical variables showed some differences. Tumors were reported in 8.9% of cases (n = 10), 6.2% of patients were classified as stage IIB (n = 8), and no final stage was documented in 26.7% of cases (n = 30).

During the study period, 7.14% (8 of 75 cases) exhibited lymphadenopathy on physical examination, which was confirmed via imaging in four patients, and 3.5% (n = 4) presented with other concomitant lymphomas. In addition, the overall mortality rate was 12.5% (n = 14), with 10 patients dying from lymphoma, 1 from COVID-19, and 3 from causes unrelated to the disease. Hematologic involvement confirmed by flow cytometry was documented in one patient.

The most frequently observed histologic features at the time of diagnosis were epidermotropism in 100% of patients with records (n = 48), Pautrier's microabscesses (5 of 43 cases), atypical lymphocytes (5 of 43 cases), and folliculotropism in 6.25% (7 of 37 cases). Mycosis fungoides was CD4+ in 50 cases (44.6%) and CD8+ in 35 cases (31.2%); however, in 27 cases no T-cell immunohistochemical profile information was obtained. Eigtheen cases (16.0%) presented the CD30+ marker (Table 4).

**Table 4:** Complementary studies and histopathological features.

	Number of cases (%)		
Variable	Yes	No	No data
Adenopathies on imaging studies	4 (3.5)	4 (3.5)	104 (92.8)
**Laboratory tests:**			
Elevated lactate dehydrogenase (LDH)	3 (2.7)	8 (7.1)	101 (90.2)
Blood count with eosinophilia	0 (0.0)	7 (6.3)	105 (93.7)
Abnormal peripheral blood smear	1 (0.89)	4 (3.5)	107 (95.5)
**Histopathological characteristics:**			
Epidermotropism	48 (42.8)	0 (0.0)	64 (57.1)
Pautrier's microabscesses	4 (3.5)	46 (41.1)	62 (55.3)
Atypical lymphocytes	38 (33.9)	0 (0.0)	74 (66.1)
Mitotic activity	3 (2.7)	32 (28.5)	77 (68.7)
Folliculotropism	7 (6.3)	30 (26.7)	75 (66.9)
CD4+ marker	50 (44.6)	15 (13.4)	47 (41.9)
CD8+ marker	35 (31.2)	24 (21.4)	53 (47.3)
CD7+ marker	12 (10.7)	46 (41.1)	54 (48.2)
CD30+ Marker	18 (16)	30 (26.7)	64 (57.1)

Note: Data collected by author in 2021.

Narrowband UVB phototherapy (NB-UVB) was the most common first-line treatment with 27.7% (n = 31), followed by UVA phototherapy + psoralens (PUVA) with 25.8% (n = 29), and topical corticosteroids with 25% (n = 28). UVA-1 phototherapy, topical imiquimod, and combinations of NB-UVB phototherapy with topical corticosteroids, interferon, or imiquimod, as well as PUVA with methotrexate, were also utilized. The median response time to treatment was 8 months (IQR 5‒14), with 31.25% (n = 35) achieving complete response, 33.04% (n = 37) achieving partial response, and 11.61% (n = 13) achieving no response. No information regarding clinical response was reported in 24.1% (n = 27). Table 5 contains information regarding second and third-line therapies.

**Table 5:** Lines of treatment implemented, type of treatment, and response time.

Item	First line, frequency (percentage)		Second line, frequency (percentage)		Third line, frequency (percentage)	
**Treatment**	NB-UVB Phototherapy	31 (27.67)	No data	38 (33.93)	No data	71 (63.39)
	PUVA Phototherapy	29 (25.89)	Topical corticosteroid	20 (17.86)	Topical corticosteroid	5 (4.46)
	Topical corticosteroid	28 (25)	PUVA phototherapy	12 (10.71)	NB-UVB phototherapy	2 (1.79)
	UVA1 Phototherapy	3 (2.67)	Topical Imiquimod	11 (9.82)	Interferon	2 (1.79)
	Topical corticosteroid and NB-UVB	3 (2.67)	NB-UVB Phototherapy	6 (5.36)	Doxorubicin	2 (1.79)
	Topical imiquimod	2 (1.79)	Topical corticosteroid and PUVA	6 (5.36)	PUVA phototherapy	1 (0.89)
	PUVA and methotrexate	2 (1.79)	Methotrexate	4 (3.57)	Methotrexate	1 (0.89)
	NB-UVB and interferon	2 (1.79)	PUVA and methotrexate	2 (1.79)	Topical corticosteroid and PUVA	1 (0.89)
	NB-UVB and imiquimod	2 (1.79)	PUVA and interferon	2 (1.79)	NB-UVB and methotrexate	1 (0.89)
	Others	8 (7.14)	UVA1 Phototherapy	2 (1.79)	UVA1 and interferon	1 (0.89)
	No data	2 (1.79)	Radiation therapy	1 (0.89)	Interferon and isotretinoin	1 (0.89)
			Oral retinoid	1 (0.89)		
			Topical corticosteroid and NB-UVB	1 (0.89)		
			UVA1 and interferon	1 (0.89)		
			PUVA and imiquimod	1 (0.89)		
			NB-UVB and imiquimod	1 (0.89)		
			Vorinostat and PUVA	1 (0.89)		
			Methotrexate and oral corticosteroids	1 (0.89)		
**Type of response to treatment**	Partial	37 (33.04)	No data	57 (50.89)	No data	77 (68.75)
	Complete	35 (31.25)	Complete	21 (18.75)	Partial	5 (4.46)
	No data	27 (24.11)	Partial	13 (11.61)	Complete	4 (3.57)
	Null	13 (11.61)	Null	5 (4.46)	Null	2 (1.79)
	Worsening	0 (0.00)	Worsening	4 (3.57)	Worsening	0 (0.00)
**Response time to treatment, median (IQR)**		8 ( 5‒14)	**Response time to treatment, median (IQR)**	4 (0.00‒10)	**Response time to treatment, median (IQR)**	0 (0.00‒0.75)

Note: Data collected by author in 2021.

Progression to an advanced stage (>IIB) occurred in 9 out of 82 cases in which the initial and final stages could be determined during follow-up, accounting for 8.04% of the total study population. In 6 of 82 cases, the median time to progression was 46 months. In the subgroup of patients who progressed, 55.5% were men (5 of 9 cases), with a median age of 59 for both sexes (IQR 43‒96). Moreover, the folliculotropic variant was observed in 44.4% (4 of 9 cases), association with other lymphomas in 22.2% (2 of 9 cases), positivity for immunohistochimistry (IHQ) CD30+ biomarker in 44.4% (4 of 9 cases), hematologic involvement in one case (11.1%), and adenomegaly in one patient (11.1%). Five of the 9 patients who progressed died, 4 from the disease and 1 from an unrelated cause.

## Discussion

After analyzing the sociodemographic characteristics identified in the present study, the median age was found to be 53 years, which is consistent with the literature, which generally indicates that this condition is more common in the sixth decade of life.^4^ Furthermore, males are the most commonly affected sex (56%), with male/female ratios of up to 2.1:1,^4^, ^7^ which is consistent with the PROCLIPI (PROspective International Cutaneous Lymphoma Prognostic Index) cohort study, which shows a male/female ratio of 1.7:1.^4^

In the present study, the time elapsed between lesion onset and diagnosis was 60 months (IQR 24‒96). This finding is longer than the time periods reported in a global cohort, which showed a delay of 36 months,^4^ and in a Brazilian study, which found a delay of 51.08 months,^8^ a figure closer to our Latin American population and under similar sociodemographic conditions. This could be due to a lack of knowledge about the disease in primary care settings, limited access to health services, and clinical polymorphism in the disease.

Furthermore, in this study, the most common clinical variant was classic (67.9%; n = 76), followed by folliculotropic (16%; n = 18). This figure is comparable to the 17.8% found in the PROCLIPI study in early stages of MF,^4^ but it is higher than the 10% with the folliculotropic variant found in a Dutch cohort of 306 patients.^9^

In the present study, the authors found the hypopigmented variant in 10.71% (n = 12) of participants, with a median age of 53 years (IQR 26.5‒46), similar to a Brazilian study conducted by Amorim et al. However, their cohort included pediatric patients, in whom this subtype of the disease is more common, unlike this study where the authors did not include this age group.^8^ The present study identified patients in their seventh decade of life with this variant, indicating a possible late presentation of this variant in the South American population. Additionally, the authors included patients with a variety of phototypes, which is contrary to what was reported in the PROCLIPI study^4^ and other studies, including Asian cohorts, which show a higher prevalence of hypopigmented MF in high phototypes.^10^

The most widely used first-line treatment in the present study was NB-UVB phototherapy, followed by PUVA phototherapy, and topical corticosteroids. This is contrary to what was found in the analysis of treatment for early-stage mycosis fungoides in the PROCLIPI study,^11^ in which the most commonly used therapies were topical steroids (39.2%), followed by PUVA (18.5%), and UVB (18.4%), finding overall response rates of 68%, 83%, and 74%, respectively.

NB-UVB phototherapy showed a complete response rate of 35.5% (11 of 31 cases), a partial response rate of 22.6% (7 of 31 cases), and a null response rate of 6.5% (2 of 31 cases), with a median time to observe the best response of 10.5 months (IQR 4.5‒15). In turn, PUVA phototherapy led to a complete and partial response rate of 37.9% (11 of 29 cases) and a null response rate of 6.89% (2 of 29 cases), with an overall median response time of 8 months (IQR 5‒13).

Topical corticosteroids demonstrated a complete disease response rate of 25% (7 of 28 cases), a partial disease response rate of 25% (7 of 28 cases), and a null response rate of 17.9% (5 of 28 cases), with a median time to observe the best response of 7 months (IQR 4‒10). These rates are lower than those reported in the PROCLIPI study on early-stage treatment.^11^ Other studies reported with the use of NB-UVB therapy a complete remission rate of 54.2% to 81%, with a mean relapse interval of 2 to 66 months, and response rates of 50% to 88% in stage IA with NB-UVB. With PUVA phototherapy, response rates oscillated between 50% to 60% in stage IB.^12^, ^13^

Concerning prognosis and overall disease progression rate of the patients included in this study, it was found that 8.04% (n = 9) progressed from an initial stage (IA‒IIA) to more advanced stages (> IIB) in a 10-year follow-up period, which is lower than the figure reported by Quaglino et al., who describe a progression rate of 29.7% in a 14.5-year follow-up period.^14^ Similarly, a retrospective study conducted in Brazil by Amorim et al. estimated a progression rate of 29.4% with a 7.85-year follow-up.^8^ The lower percentage of progression found in the present study could be explained by the representation of patients in very early stages of the disease (64% in stage IA), together with a subgroup of patients with the hypopigmented variant (10.7%) who have a very favorable prognosis in general.

When characterizing the patients in the present study who experienced progression, it was found that age (median 59 years, IQR 43‒96) acted as an adverse prognostic factor for progression (p = 0.026), as mentioned by Scarisbrick et al.^5^ Other potential prognostic factors mentioned in the literature and found in this subgroup were: The folliculotropic variant was observed in 44.4% of patients who experienced progression, which is categorized in the literature as having a poor prognosis due to a higher risk of advancing to advanced stages^9^ and a lower overall survival rate. A Colombian study by Pérez et al. reported a 5-year overall survival of 62% in patients with folliculotropic MF and 40% in patients with the same variant but advanced disease.^15^

The presence of CD30 expression, found in 44.4% of patients who progressed, is a significant finding since it has been reported in multiple studies as an important histopathological characteristic associated with a higher risk of disease progression and increased mortality rates.^7^

Lymphadenopathy was observed in 11.1% of patients, confirming its higher association with advanced stages of the disease and patients with Sézary Syndrome, rather than those in early stages.^16^

The presence of concurrent lymphomas at diagnosis was noted in 22.2% of cases. It has been observed in the literature as a risk factor for stable disease evolution or progression to advanced stages. Additionally, having a diagnosis of Mycosis Fungoides is a risk factor for developing other types of cutaneous lymphomas, as explained in a study conducted in the United States by Jawed et al.^17^

When comparing the present study’s data with the retrospective cohort study conducted by Miyashiro et al., involving 727 patients, it was found that the diagnosis of Sezary Syndrome, age ≥60 years, folliculotropic variant, erythrodermic variant, advanced clinical stage, elevated levels of lactate dehydrogenase, and presence of large cell transformation were associated with a worse prognosis of the disease.^18^

Finally, in this study, overall mortality was 12.5% (n = 14), with 10 patients dying from lymphoma, one from COVID-19, and three from causes unrelated to the disease. These findings are consistent with the literature, which states that early MF has a median 5-year survival rate of more than 80%, compared to advanced-stage disease, which has a predicted 5-year survival rate of around 50%.^11^ A recent meta-analysis found a 5-year survival rate of 85.8% for stage IB, 62.2% for stage IIB, 59.7% for stage IIIA, 54% for stage IIIB, 52.5% for stage IVA1, 34% for stage IVA2, and 23.3% for stage IVB.^19^

## Conclusion

Early mycosis fungoides is a disease with a good prognosis in most patients, with a progression rate of 8% (n = 9).

## Financial support

None declared.

## Authors' contributions

Santiago Andrés Ariza Gómez: The study concept and design; writing of the manuscript or critical review of important intellectual content; effective participation in the research guidance; intellectual participation in the propaedeutic and/or therapeutic conduct of the studied cases; critical review of the literature; final approval of the final version of the manuscript.

Paula Alejandra Dubeibe Abril: The study concept and design; data collection, or analysis and interpretation of data; statistical analysis; writing of the manuscript or critical review of important intellectual content; data collection, analysis, and interpretation; effective participation in the research guidance; final approval of the final version of the manuscript.

Oscar Enrique Niebles Sincelejo: The study concept and design; data collection, or analysis and interpretation of data; statistical analysis; writing of the manuscript or critical review of important intellectual content; data collection, analysis, and interpretation; effective participation in the research guidance; final approval of the final version of the manuscript.

Henry Santiago Leal Reina: The study concept and design; data collection, or analysis and interpretation of data; statistical analysis; writing of the manuscript or critical review of important intellectual content; data collection, analysis, and interpretation; effective participation in the research guidance; final approval of the final version of the manuscript.

## Conflicts of interest

None declared.
